# The Effectiveness of a Digital Mental Fitness Program (Positive Intelligence) on Perceived Stress, Self-Compassion, and Ruminative Thinking of Occupational Therapy Undergraduate Students: Longitudinal Study

**DOI:** 10.2196/49505

**Published:** 2024-10-07

**Authors:** Shermain Puah, Ching Yee Pua, Jing Shi, Sok Mui Lim

**Affiliations:** 1 SIT Teaching and Learning Academy (STLA) Singapore Institute of Technology Singapore Singapore; 2 Health and Social Sciences Singapore Institute of Technology Singapore Singapore

**Keywords:** mental health, students, digital wellness, mobile health (mHealth), perceived stress, self-compassion, rumination

## Abstract

**Background:**

Health care students often endure numerous stressors throughout their undergraduate education that can have lasting negative effects on their mental well-being. Positive Intelligence (PQ) is a digital mental fitness program designed to enhance self-mastery and help individuals reach their potential by strengthening various “mental muscles.”

**Objective:**

This study aims to evaluate the effectiveness of a 6-week app-delivered PQ program in reducing perceived stress, increasing self-compassion, and decreasing rumination tendencies among health care undergraduates. We hypothesized that students would show reductions in perceived stress, increases in self-compassion, and decreases in rumination tendencies by the end of the PQ program, compared with their preprogram scores. We adopted an exploratory approach for the 5-month follow-up due to the limited research consensus on the sustained effects of app-based programs over varying periods.

**Methods:**

The PQ program includes weekly hour-long videos, weekly group meetings, and daily 15-minute app-guided practices. Participants were first-year students from the occupational therapy program at a university in Singapore. Participants completed surveys measuring self-compassion, perceived stress, and rumination levels before and after the PQ program, and again at a 5-month follow-up. Data were analyzed using repeated measures ANOVA to assess differences across the pretest, immediate posttest, and follow-up posttest.

**Results:**

Out of 87 students enrolled in the study, the final sample consisted of 64 students (n=47, 73%, female; mean age 23 years, SD 5.06 years) with complete data. At the end of the 6 weeks, students exhibited significant increases in self-compassion (before the intervention: mean 3.07, SD 0.35; after the intervention: mean 3.34, SD 0.35; *P*<.001) and reductions in rumination tendencies (before the intervention: mean 3.57, SD 0.40; after the intervention: mean 3.27, SD 0.34; *P*<.001). However, no significant change in perceived stress levels was observed (before the intervention: 2.99, SD 0.14; after the intervention: mean 2.97, SD 0.16; *P*=.50). These effects were not influenced by the daily app-based practice of PQ exercises, and there were no sustained effects on self-compassion (mean 3.17, SD 0.27; *P*=.09) or rumination tendencies (mean 3.42, SD 0.38; *P*=.06) at the 5-month follow-up. Additionally, there was a significant increase in perceived stress at follow-up (mean 3.17, SD 0.21; *P*<.001) compared with pre- and postintervention levels.

**Conclusions:**

The PQ program did not directly alter stress perceptions but may have reframed students’ automatic negative thought processes, increased their awareness of self-sabotaging behaviors, and enhanced their self-compassion while reducing ruminative thinking. These findings highlight the importance of self-awareness for students’ well-being. Students can benefit from practices such as mindfulness and peer discussions to enhance self-compassion and reduce rumination. Educators trained in the PQ program can foster a supportive environment that encourages self-compassion, challenges negative self-talk, and helps students manage stress.

## Introduction

### Background

A growing body of literature highlights the increasing levels of stress, anxiety, and psychological distress experienced by undergraduate students in health care disciplines [1]. Common stressors during their education include clinical workload, expectations during clinical placements, academic program demands, insufficient support to manage both clinical and academic responsibilities, and the emotional toll of working with patients suffering from severe conditions [2-4]. For some students, the transition to university itself can be overwhelming [5].

The psychological and emotional distress resulting from these challenges and demands can significantly impact the mental well-being of health care students, not only during their education but also potentially extending into their transition into the workforce as health care professionals [6,7]. Given the potential impact on work-relevant functioning, helping students develop coping strategies to mitigate these effects throughout their professional lives is an important area of investigation [7,8]. Therefore, efforts should focus on teaching self-care and promoting well-being at the undergraduate level, supporting students in building habits that will help them manage stress and challenges effectively.

### Positive Intelligence

Positive Intelligence (PQ), a method developed by Chamine [9] at Stanford University as a personal development tool, aims to build mastery over one’s mind to fulfill one’s potential (ie, mental fitness). The PQ program is designed to fit into the busy schedules of modern individuals by offering bite-sized practice exercises and videos over a short period (6 weeks). Although it has been applied to MBA (master of business administration) students, athletes, executive teams, and chief executive officers [10], its use in the fast-paced and demanding health care setting remains largely unexplored.

According to Chamine’s findings [9], throughout life, every experience we have helps to develop and solidify certain automatic mental attitudes and habits. These mental habits can either work in our favor or against us, placing us in a state of “thriving” or “survival.” Two opposing states of mind, namely, the “sage” and the “saboteurs,” are in a constant tug of war, as they are wired for very different primary purposes. The “sage” is wired for creativity, empathy, expanding perspectives, and fostering positive emotions. By contrast, the “saboteurs” trigger survival instincts, narrow perspectives, and generate negative emotions. The risk of remaining under the influence of one’s “saboteurs” is the missed opportunities that arise in challenging situations due to an inability to see beyond the negativity. The goal of PQ is to build mental fitness by strengthening “mental muscles”—choosing “sage” responses and intercepting the tendencies of one’s “saboteurs.”

The PQ program is unique in that it goes beyond training mindfulness. It incorporates additional components such as increasing awareness of one’s inner critics (or “saboteurs”), learning to adopt a perspective that sees opportunities and gifts in challenges (the “sage” perspective), and participating in group support through “Pod” meetings. These elements work together to create a comprehensive and holistic experience. The PQ program spans 6 weeks and is primarily delivered through a mobile app, though some components may also be accessed via a web browser. A “muscle” score is cumulatively logged in the app whenever an individual practices the “self-command muscle” (ie, the ability to self-regulate thoughts and feelings), the “saboteur interceptor muscle” (ie, the ability to intercept and disarm the false messages of “saboteurs”), or the “sage muscle” (ie, the ability to use wisdom and insight to handle challenges) over the 6 weeks [11]. The implementation of the PQ program in this study is detailed in the “Methods” section.

According to Positive Intelligence [11], the PQ program has been utilized by over 500,000 individuals from diverse backgrounds and ages, across as many as 50 countries. However, there is limited scholarly research assessing the effectiveness of the PQ program in providing students with the mental fitness needed to manage stressors during their undergraduate education. We propose that the PQ program may effectively enhance health care students’ self-compassion, reduce perceived stress, and decrease rumination tendencies. These variables—perceived stress, self-compassion, and rumination—were chosen for this study due to their critical roles in the psychological well-being of health care students and their importance in protecting against emotional distress [12-14]. Additionally, these variables align with the goals of the PQ program. The following section discusses the relevance of these variables in relation to the program and overall well-being.

### Perceived Stress

According to Lazarus [15], perceived stress is defined as a subjective emotional experience in which an individual feels that the demands placed upon them exceed their ability to cope. An individual’s perception of a stressful event is crucial, as it influences how uncontrollable and overwhelming they perceive the situation to be [16]. Among university students, elevated stress levels are more common in health care students compared with their non–health care counterparts, who are also at a higher risk of developing mental health issues [17].

Stress management is identified as a key area of impact when individuals practice PQ and learn to intercept their “saboteurs” and activate their “sage” [11]. According to research conducted by the Positive Intelligence company [11], stress is viewed as a negative emotion resulting from the habitual activation of one’s “saboteurs.” These “saboteurs” are responsible for generating many negative emotions. While these emotions can be useful in alerting individuals to dangers or issues when activated briefly, they impair one’s ability to think clearly and make optimal decisions [11]. By practicing the act of focusing attention on a particular sensation or object (the “self-command muscle”) and intercepting one’s inner critics (the “saboteur interceptor muscle”), individuals gain greater control over their mind and thoughts. This process enables them to adopt a more effective perspective (the “sage perspective”).

### Self-Compassion

Self-compassion involves extending empathy and support to oneself during times of suffering. Neff [18,19] conceptualized self-compassion as comprising 3 core components: kindness toward oneself versus self-judgment, a sense of common humanity versus isolation, and mindfulness versus overexaggeration when dealing with painful experiences. Our inner critics often lead us to harbor negative messages about ourselves, resulting in self-evaluation that is harsh and unkind. With self-compassion, we adopt a compassionate stance toward ourselves, allowing us to view our sufferings, lives, and experiences from a broader and more neutral perspective [20]. Existing literature consistently reports that higher levels of self-compassion are strongly associated with a lower risk of mental health issues and greater psychological flourishing [20].

The ability to intercept one’s inner critics is essential for achieving greater self-acceptance and self-compassion. According to PQ, one prominent “saboteur” is the “judge.” The “judge” manifests unconsciously when we evaluate ourselves, others, and the situations we encounter [9]. Although the primary role of the “judge” is to discern potentially harmful scenarios, habitual judgment can lead us to judge everyone and everything unconsciously, including ourselves, in an attempt to gain a sense of security or control [21]. This can eventually lead to various issues, such as negative impacts on working relationships and increased isolation. Therefore, becoming aware of the key inner critic, the “judge,” and practicing intercepting its negative evaluations of oneself, others, and external events are believed to enhance self-compassion and empathy [22].

### Rumination

Rumination is defined as the process of being in a negative affective state and repeatedly thinking about the causes, signs, meanings, and consequences of that state [23]. This process is typically self-referential and past-oriented, with individuals continually reflecting on their past to understand the origins and causes of their current situation and relating it to their present self [24,25]. Research shows that rumination is strongly correlated with negative mood, as well as anxiety and depressive symptoms [26-28].

Rumination exemplifies how our mind can engage in a nondeliberate thinking process. By contrast, PQ emphasizes practicing mindfulness and the “self-command muscle” [11]. This approach involves a deliberate process where attention is focused on being present, rather than allowing the mind to wander during rumination. Indeed, mindfulness is believed to disrupt the fixation on negative thoughts, which in turn alleviates rumination [29]. Additionally, becoming aware of our unconscious judgments of ourselves, others, and events, and practicing intercepting these negative evaluations by the “judge saboteur,” may lead to a reduction in self-referential rumination [22].

### Existing Research

Given the current lack of scholarly literature examining the effects of the PQ intervention on psychological and emotional factors, this study also draws on existing research investigating similar interventions. Specifically, it references studies on mindfulness-based mobile apps and their effects on perceived stress, self-compassion, and rumination [13,14,30-32]. The selection of perceived stress, self-compassion, and rumination as variables in this study is justified by their significant impact on the mental well-being of health care students and their alignment with the objectives of the PQ program. By focusing on these variables, the study aims to provide comprehensive insights into the program’s potential to enhance mental fitness and well-being, thereby equipping students with the tools needed to thrive in their demanding educational and professional environments.

Recent literature has recognized the inverse relationship between mindfulness and rumination. Goldberg et al [33] evaluated the effects of mindfulness training, combined with self-compassion practice through introspection, using an app-delivered mindfulness meditation program. The study found that students’ self-reported levels of empathy, self-compassion, self-reflection, and rumination improved following the intervention. In a waitlist control experimental study, Huberty et al [34] assessed the impact of an 8-week mindfulness meditation mobile app on university students’ perceived stress, mindfulness, self-compassion, and other health behaviors. The authors reported a significant reduction in stress and an increase in self-compassion immediately following the intervention, with these effects sustained at a 1-month follow-up. However, the short follow-up period limits the ability to draw long-term conclusions.

Similar to the PQ mobile app, the study by Huberty et al [34] used the Calm app (Calm.com, Inc.) mobile app, which provided users with daily guided (or unguided) mindfulness meditation exercises. Therefore, we can similarly expect that participants in the PQ program might experience a reduction in stress and increases in self-compassion from the 15 minutes of daily mindfulness practice. The Calm mobile app primarily aims to provide mindfulness meditation to users. Unlike the fixed 6-week program structure of PQ, the aforesaid app allows users to choose from various topics or weekly series targeting different goals (eg, managing stress or increasing self-esteem). Additionally, the exercises vary in length, instruction, and mindfulness principles.

Previous studies on web-based or app-based mindfulness interventions have also reported similar reductions in stress and anxiety after several weeks [13,30-32]. For example, Orosa-Duarte and colleagues [14] compared the effects of a mindfulness-based mobile app with those of an in-person mindfulness training program on anxiety, empathy, self-compassion, and mindfulness among a group of health care students. The authors found that delivering mindfulness training through a mobile app can be as effective as in-person training in reducing anxiety and increasing self-compassion among health care students. These results align with recent systematic reviews and meta-analyses, which have shown that online mindfulness interventions are beneficial for enhancing self-compassion [35] and reducing stress, depression, and anxiety-related symptoms after intervention [36].

Using a chatbot developed based on the self-help approach by Chamine [9], Kroon [37] used this intervention to help health care workers identify and become aware of their inner critics (saboteurs) and address these critics to enhance self-compassion and reduce compassion fatigue. The author reported that a common theme in interviews with participants was an increased awareness of their inner critics and the negative impact these critics had on their self-compassion and self-kindness. Similarly, a previous study investigating the effects of self-compassion meditation in caregivers found that increasing awareness of one’s inner judgmental critic led to a greater recognition of the need to show compassion toward both oneself and others [38].

The PQ program addresses a common barrier to engagement in mobile health (mHealth) interventions—lack of social connectedness—through its “Pod” group meetings. A review by Borghouts et al [39] highlighted numerous studies showing that participants’ sense of social connectedness (eg, regular interactions with peers or a personal therapist) enhances user engagement. When participants could emotionally connect with peers by openly sharing problems or relating to others’ experiences, their motivation to continue using mental health apps increased [40,41].

### Study Objectives

This study aims to evaluate the efficacy of the 6-week PQ program, which includes increasing awareness of inner critics (saboteurs) in one’s personal life, training intentional and deliberate control of one’s mind and thoughts (self-command muscle), intercepting inner critics (saboteur interceptor muscle), and adopting a different perspective by transforming difficult situations or challenges into specific gifts or opportunities (sage muscle). We will measure health care students’ self-compassion, perceived stress, and rumination tendencies before and after the PQ program, and again at a follow-up a few months later, to assess the program’s efficacy.

This study addresses the following research question (RQ): “What is the effect of undergoing the 6-week PQ program on health care students’ perceived stress, self-compassion, and rumination tendencies, measured before and after the program?” (RQ1). Based on existing findings, we expect that students will show decreases in perceived stress, increases in self-compassion, and decreases in rumination tendencies immediately after completing the PQ program, compared with their scores before the program. However, there has been less consensus on the sustained effects of app-based programs at follow-up after varying periods [26]. Therefore, we pose the following RQ: “To what extent does undergoing the 6-week PQ program benefit health care students’ perceived stress, self-compassion, and rumination tendencies at a 5-month follow-up compared with baseline measures before the program?” (RQ2).

A subquestion is as follows: “To what extent do changes in self-compassion, perceived stress, and rumination tendencies depend on whether students have consistently engaged in daily practice throughout the PQ program, as indicated by their cumulative “PQ muscle” count at the end of the 6 weeks?” (RQ3). Addressing this RQ will clarify whether consistent app-based practice contributes to changes in the outcome variables. In line with previous studies on web-based or app-based mindfulness interventions [13,30-32], we recognize that the effectiveness of the PQ program for perceived stress, self-compassion, and rumination tendencies requires a conscious effort from students. Therefore, we hypothesize that students with a higher cumulative average “PQ muscle” count after the program will exhibit a greater decrease in perceived stress and rumination tendencies and a greater increase in self-compassion.

## Methods

### Sample

This study used a convenience sampling method, with participants drawn from the freshmen cohort of the occupational therapy degree program at a Singapore-based university. All 98 students were beginning their first trimester of occupational therapy studies. No exclusion criteria were applied, so all students in the cohort were eligible as potential participants. As the 6-week PQ program was integrated into a module, no additional incentives were provided for completing the program or participating in the research.

### Intervention: PQ Program

**Figure 1:**
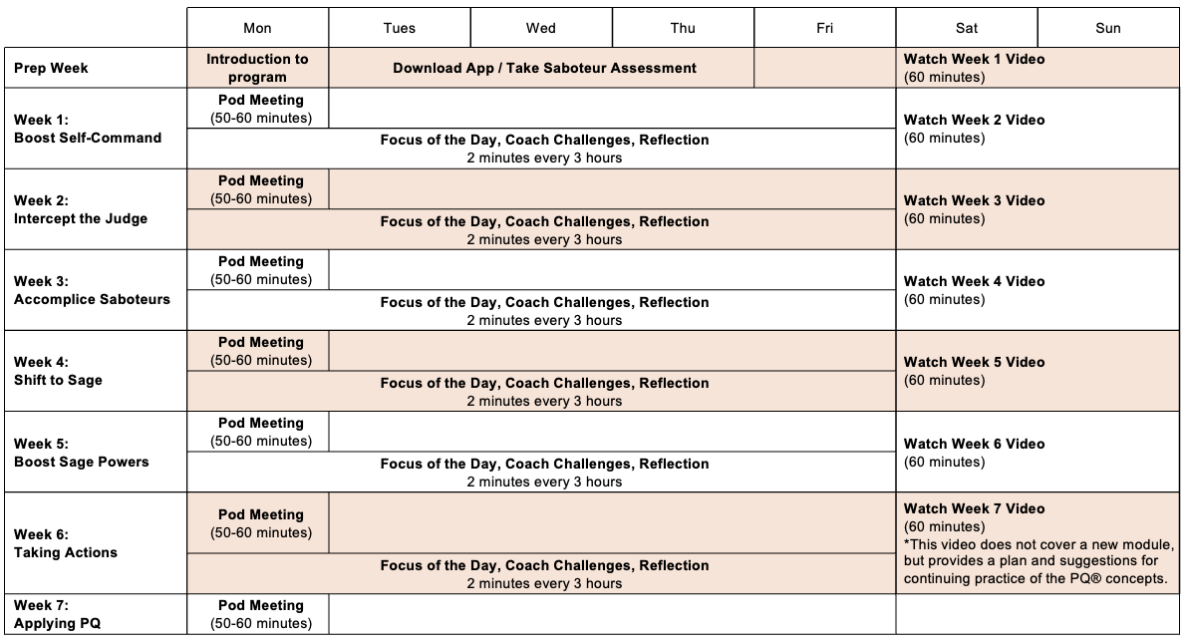
Details of the PQ program. Adapted from Positive Intelligence, LLC (personal communication, June 14, 2022). PQ: Positive Intelligence.

The PQ program consists of 3 core modules spread over 6 weeks and is completed individually on the PQ mobile app at the participant’s convenience, with the exception of scheduled small group (Pod) meetings. The program includes 15 minutes of daily practice (eg, “Focus of the Day” or “Coach Challenges”); weekly hour-long videos, which are broken down into short segments, with each segment lasting between 3 and 15 minutes; and weekly 45-minute small group meetings (Figure 1). The 15 minutes of daily practice can be divided into several practice blocks (PQ reps) lasting from 10 seconds to a few minutes [42]. These blocks involve brief mindfulness practices [43], which may include focusing attention on a specific sensation or object, such as breathing, tactile sensations, or sounds, or bringing into awareness what one is experiencing as they arise in the present moment.

In the first week, participants focus on the concept of the “self-command muscle,” practicing the development of intentional and deliberate control over their minds and thoughts through “PQ reps.” As participants strengthen their “self-command muscle,” they can then integrate “PQ reps” with cognitive reframing techniques to effectively intercept their “saboteurs” and activate “sage” responses [42].

In the second and third weeks, participants focus on the “saboteur interceptor muscle,” practicing how to intercept and shut down their inner critics (saboteurs). During the third week, the app customizes content based on each participant’s top-ranking “saboteur” from the Saboteur Assessment, providing targeted exercises to further develop their “saboteur interceptor muscle.” Inner critics (or saboteurs) are defined as internal voices that fuel negative messages and evaluations of oneself, typically developed unconsciously over time [9]. These inner critics are shaped and reinforced by interactions with close others or people in one’s environment; experiences of criticism, neglect, or inconsistency in these interactions can lead individuals to form negative self-evaluations (eg, feeling inadequate, unworthy, or useless) [9,44].

Finally, in the fourth to sixth weeks, participants focus on developing the “sage muscle,” practicing how to adopt the “sage” perspective by converting difficult situations or challenges into specific gifts or opportunities. For example, the “Three Gifts” technique helps individuals transform challenges or disappointments into valuable opportunities by leveraging them to cultivate personal strengths, gain new insights, or initiate inspired actions as a direct outcome of adversity [42].

### Procedure

All students were instructed to download the PQ app from Google Play (Alphabet Inc.) or Apple App Store (Apple Inc.). The rationale and aims of the PQ program were explained to the students through onboarding materials, which included an overview of the program, an introduction to the app’s functions, and details on how the “Pod” groups should operate. Students were assigned to small groups of 5 or 6, known as “Pods.” They were encouraged to watch the weekly videos by Monday each week in preparation for their virtual “Pod” discussions on Tuesday, which were scheduled for an hour after the module lecture. Students were also encouraged to practice for at least 15 minutes a day using the “Focus of the Day” and “Coach Challenges” features and to log their practice time on the app. Before officially starting the program, students were required to complete the Saboteur Assessment online [45,46], which ranks the dominance of each of the 9 saboteurs [21] in their personal lives.

In addition to the Saboteur Assessment, students completed an online survey questionnaire measuring perceived stress, self-compassion, and rumination as a baseline (pretest). Before taking the survey, students were first asked to consent or decline participation in the study. They were presented with an information sheet detailing the study’s purpose, process, and the differences between research participants and nonparticipants, followed by an informed consent form.

Participants completed the same survey questionnaire immediately after the intervention (immediate posttest). To assess the sustained effects of the program on perceived stress, self-compassion, and rumination levels, participants took the questionnaire again 5 months after completing the program (follow-up posttest). During this follow-up, participants were also asked how frequently they continued to use the PQ mobile app since the completion of the 6-week PQ course.

### Measures

#### Measurement of Stress, Self-Compassion, and App Usage Over 6 Weeks

A survey questionnaire measuring perceived stress, self-compassion, and rumination was administered at 3 time points. App usage was measured objectively using the PQ app, which logs a cumulative “muscle” score whenever an individual practices the “self-command muscle,” “saboteur interceptor muscle,” or “sage muscle” over the 6 weeks. PQ reps are automatically recorded on the app as an indicator of use. Users also have the option to manually enter their mindfulness practice if they do not use the standard app exercises.

#### Perceived Stress

The 14-item Perceived Stress Scale (PSS-14; [47]) assesses the frequency with which individuals have experienced perceived stress over the past 2 months (eg, “How often have you felt that difficulties were piling up so high that you could not overcome them?”). All items are rated on a 5-point Likert scale (1=never to 5=always), with higher scores indicating greater perceived stress experienced over the past 2 months. Cronbach α for this sample was >0.84 at all measurement time points, indicating the high reliability of the measure. This is consistent with existing evidence demonstrating that the PSS-14 is a reliable and valid measure of perceived stress across various samples of university students [48-50].

#### Self-Compassion

The 12-item Self-Compassion Scale–Short Form (SCS-SF; [51]) assesses how respondents view their actions toward themselves during difficult times (eg, “When times are really difficult, I tend to be tough on myself”). Items are rated on a 5-point scale (1=strongly disagree to 5=strongly agree), with higher scores indicating greater self-compassion. Cronbach α for this sample ranged from 0.78 to 0.83 at all measurement time points, indicating the high reliability for this measure in our study. This aligns with previous findings that demonstrate the 12-item SCS-SF’s robust psychometric properties, including high internal reliability [52,53] and strong face, content, convergent, and divergent validity [51,54].

#### Rumination

The 12-item Rumination subscale of the Reflection Rumination Questionnaire (RRQ-Rum; [55]) was used to assess rumination (eg, “Long after an argument or disagreement is over, my thoughts keep going back to what happened”). Items are rated on a 5-point scale (1=strongly disagree to 5=strongly agree), with higher scores indicating greater rumination tendencies. Cronbach α for this sample was >0.88 at all measurement time points, demonstrating the high reliability of the measure. This is consistent with previous studies that report high internal reliability [56,57] and robust convergent and divergent validity [58,59].

### Data Analysis

This study aims to evaluate the efficacy of the 6-week PQ program on health care students’ perceived stress, self-compassion, and rumination tendencies. To assess differences in perceived stress, self-compassion, and rumination scores across the pretest, immediate posttest, and follow-up posttest, a repeated measures ANOVA was conducted. The data set was cleaned and prepared for analysis, including only data from participants who completed measurements at all 3 time points. Before conducting the analyses, Shapiro-Wilk tests were performed to assess the assumption of normality, and Mauchly tests were used to check for violations of the assumption of sphericity. This analysis will help determine whether there are statistically significant differences in students’ perceived stress, self-compassion, and rumination tendencies from pre-intervention to postintervention and at follow-up 5 months later. For each outcome variable, pairwise comparisons using the Tukey honestly significant difference (HSD) test were conducted when significant differences were observed.

To address RQ3, which asks, “To what extent does the change in self-compassion, perceived stress, and rumination tendencies scores depend on whether students have consistently engaged in daily practice throughout the PQ program, as indicated by their cumulative ‘PQ muscle’ count at the end of the six weeks?” A linear regression analysis with an interaction term was conducted to test for moderation effects. Posttest scores for each of the outcome variables—perceived stress, self-compassion, and rumination—were regressed on their respective pretest scores. In each linear regression model, the cumulative PQ muscle count was included as a moderator.

### Ethical Considerations

Ethical approval for data collection was obtained from the Institutional Review Board at the Singapore Institute of Technology (approval number 2022157). All students in the cohort were enrolled in the PQ program but had the option to decline consent for their data to be used for research purposes. The first author (SP), a nonteaching staff member, informed students of the opportunity to participate in the research study both during lecture time and via email. Informed consent was obtained from participants before their involvement in the study and students were assured that their data would be used solely for research purposes. Research data were pseudonymized to protect participant confidentiality.

## Results

### Overview

Ultimately, 87 out of 98 students from the occupational therapy freshmen cohort signed the informed consent form. Of these, 76 students completed the pretest, which measured self-compassion, perceived stress, and rumination tendencies. Following the 6-week PQ program, 78 students completed the same questionnaire for the immediate posttest. At follow-up, 87 students completed the questionnaire 5 months after the completion of the 6-week PQ program (follow-up posttest).

Of these students, 64 could be matched across time, having completed the pretest, immediate posttest, and the follow-up posttest. Of the 64 students analyzed, 47 (73%) were female (mean age 23 years, SD 5.06 years). The distribution of the cohort also had a female majority, with 76 out of 98 students (78%) being female.

### Primary Outcomes

#### Perceived Stress

**Figure 2:**
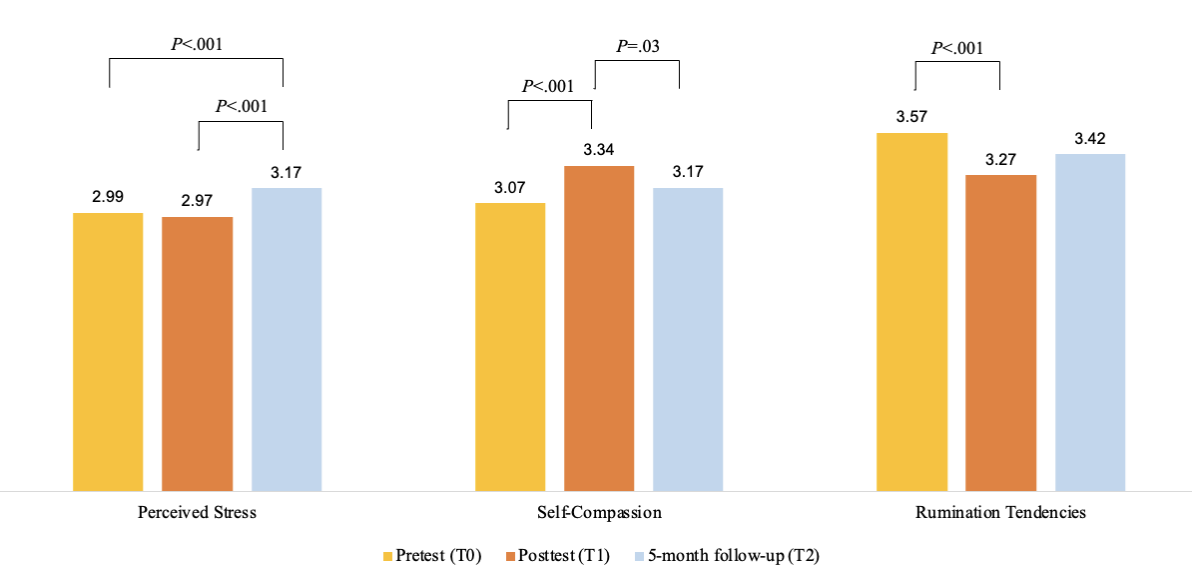
Changes in students' stress, self-compassion, and rumination from pretest to posttest to 5-month follow-up.

The assumptions of normality (Shapiro-Wilk tests: pretest, *P*=.37; posttest, *P*=.55; and after 5 months, *P*=.21) and sphericity (Mauchly test: W=0.96, *P*=.24) were met. A repeated measures ANOVA revealed a statistically significant difference in perceived stress scores across the 3 time points (*F*_2,126_=10.55, *P*<.001, η^2^*_g_*=0.05). Contrary to our hypothesis, pairwise comparisons using the Tukey HSD test showed no significant change in students’ perceived stress ratings after the 6-week PQ program (mean 2.97, SD 0.16; *P*=.50) compared with their baseline scores measured before the start of the program (mean 2.99, SD 0.14). Instead, we found a significant increase in students’ ratings of perceived stress at follow-up (mean 3.17, SD 0.21; *P*<.001) from perceived stress scores measured before and after the intervention (Figure 2).

#### Self-Compassion

The Shapiro-Wilk test confirmed that the assumption of normality was met for pretest scores (*P*=.73) but a slight departure from normality for the other time points (posttest, *P*=.02; after 5 months, *P*=.01); however, visual inspection of the Q-Q plots suggested that the deviations were minor. The assumption of sphericity (Mauchly test: W=.97, *P*=.45) was met. A repeated measures ANOVA found a statistically significant difference in self-compassion ratings across the 3 time points (*F*_2,126_=9.83, *P*<.001, η^2^*_g_*=0.05). As hypothesized, pairwise comparisons with the Tukey HSD test showed a significant increase in students’ self-compassion ratings after the 6-week PQ program (mean 3.34, SD 0.35; *P*<.001) compared with self-compassion scores before the program (mean 3.07, SD 0.35). However, at follow-up (mean 3.17, SD 0.27), self-compassion ratings were no different from that of scores before the program (*P*=.09) but were significantly less than scores immediately after the program (*P*=.03).

#### Rumination

We confirmed that the assumptions of normality (Shapiro-Wilk tests: pretest, *P*=.23; posttest, *P*=.14; and after 5 months, *P*=.57) and sphericity (Mauchly test: W=.97, *P*=.44) were met. A repeated measures ANOVA found a statistically significant difference in rumination scores across the 3 time points (*F*_2,126_=8.48, *P*<.001, η^2^*_g_*=0.04). In line with our hypothesis, pairwise comparisons with the Tukey HSD test showed a significant decrease in students’ rumination tendencies after the 6-week PQ program (mean 3.27, SD 0.34; *P*<.001) compared with rumination tendencies before the program (mean 3.57, SD 0.40). However, at follow-up (mean 3.42, SD 0.38; *P*=.06), rumination scores were no different from that of scores before the program or immediately after the program.

### App Usage

Results of the moderated regression analysis revealed no significant interaction effect between pretest scores of perceived stress, self-compassion, or rumination and PQ muscle counts on immediate posttest scores for these variables (*P*=.62 for perceived stress, *P*=.65 for self-compassion, and *P*=.42 for rumination; Table 1). This means that contrary to our hypothesis, changes in perceived stress, self-compassion, and rumination scores from pretest to posttest did not depend on whether students engaged consistently in daily app-based practices throughout the PQ program, as indicated by their cumulative “PQ muscles” logged in the app.

At the 5-month follow-up, participants were asked about their continued use of the PQ mobile app since the completion of the 6-week program. Of the 64 students who completed the follow-up survey, only 1 (2%) participant reported using the app “sometimes.” By contrast, 46 (72%) participants indicated they had “never” used the app and 17 (27%) participants indicated they had “seldom” used it.

**Table 1:** Results of the moderated regression analyses.

Regression analysis		β (SE)	*P* value
**Perceived stress**			
	Intercept	3.00 (0.04)	<.001
	Pretest perceived stress	0.67 (0.11)	<.001
	PQ^a^ muscle	0.00 (0.00)	.19
	Pretest perceived stress × PQ muscle	0.00 (0.00)	.62
**Self-compassion**			
	Intercept	3.30 (0.05)	<.001
	Pretest self-compassion	0.41 (0.08)	<.001
	PQ muscle	0.00 (0.00)	.07
	Pretest self-compassion × PQ muscle	0.00 (0.00)	.65
**Rumination**			
	Intercept	3.30 (0.06)	<.001
	Pretest rumination	0.57 (0.09)	<.001
	PQ muscle	0.00 (0.00)	.89
	Pretest rumination × PQ muscle	0.00 (0.00)	.42

^a^PQ: Positive Intelligence.

## Discussion

### Principal Findings

This study investigated whether a 6-week app-delivered mindfulness-based mental fitness program, PQ, could decrease perceived stress levels, increase self-compassion, and reduce rumination tendencies among health care undergraduates. Our findings partially support our hypotheses: by the end of the 6 weeks, students exhibited significant increases in self-compassion and reductions in rumination tendencies. However, there was no significant effect on perceived stress levels. At the 5-month follow-up after completing the PQ program, we observed that students’ self-compassion and rumination scores were no different from their baseline measures (pretest). Additionally, perceptions of stress had increased at follow-up. Furthermore, there was no evidence that more consistent daily PQ practice, as tracked through the app, resulted in greater changes in self-compassion, perceived stress, or rumination tendencies.

### Changes to Perceived Stress, Self-Compassion, and Rumination

A particularly interesting and unexpected finding was the significant benefit of the PQ program on self-compassion and rumination levels, though not on stress. The lack of an effect on stress might be attributed to the timing of the study; our sample consisted of students in their first year and first trimester of undergraduate studies. As the participants began the PQ program and completed the pretest surveys, they were also just starting their first weeks of freshman year. Some students may have taken a long break from studying before starting their degree program, so our baseline measure might not have been the most representative of their perceived stress levels. At the same time, immediate posttest surveys were distributed during a period when several assignments were due. Consequently, although our findings did not support our hypothesis, we are encouraged by the fact that students did not report a significant increase in stress immediately after completing the program, despite the rising academic workload. We recommend that future studies include a control group to better investigate the true effect of the PQ program on perceived stress, as this would provide a clearer understanding of how the PQ program functions [60].

As perceptions of stress depend on life and work demands, it is likely that the PQ program did not directly alter students’ perceptions of stress. Instead, it may have reframed students’ automatic negative thought processes, increased their awareness of potential self-sabotage, and helped them develop greater self-compassion and reduced ruminative thinking. Rumination tendencies significantly decreased from the pretest to the posttest as hypothesized, consistent with previous research indicating that app-delivered mindfulness-based practices can reduce rumination [27,29]. Additionally, we observed a significant increase in self-compassion ratings after the 6-week program. This aligns with previous studies, such as Frewen et al [61], which found that mindfulness practice enhances an individual’s ability to let go of negative automatic thoughts. As humans, it is natural to fixate on negative thoughts and struggle to break free from the cycle of rumination. Practicing the ability to “be present” in the here and now, without getting distracted or lost in repetitive thoughts about worries or situations, enhances psychological and emotional regulation and reduces automatic negative thinking [61,62].

However, if mindfulness practice alone were responsible for the intervention effects on self-compassion and ruminative thinking, we would have expected to see a notable difference in outcomes between students who logged more daily PQ exercises and those who logged fewer. Instead, the study results showed no evidence that more consistent daily PQ practice, as tracked through the app, led to greater changes in self-compassion, perceived stress, or rumination tendencies. In other words, app usage did not moderate the changes in self-compassion, perceived stress, or rumination scores between the pretest and posttest. Therefore, our findings cannot be solely attributed to mindfulness practice alone. Other aspects of the PQ program—such as the awareness gained from the Saboteur Assessment, the weekly videos, and the weekly “Pod” meetings—may have also contributed to the observed intervention effects. Moreover, it is possible that students engaged in PQ or similar mindfulness practices outside of the app, which means that the tracked app usage might not accurately reflect the total amount of practice. However, without an active control group, our study is limited in the comparisons and conclusions we can draw.

Previous research has shown that the PQ program’s focus on awareness of one’s inner critics leads to greater self-acceptance and encourages withholding judgment toward oneself and others [37,38]. This awareness fosters empathy and compassion, helping individuals recognize that self-sabotaging patterns and tendencies are often driven by underlying struggles, such as unmet needs, vulnerabilities, or past trauma [63]. Indeed, Stjernswärd and Hansson [38] found that increasing awareness of participants’ self-sabotaging inner critics led to greater compassion and acceptance toward themselves, others, and external situations. Furthermore, gaining awareness of these inner critics is essential for effectively interrupting negative thinking patterns. This approach helps reduce self-referential rumination tendencies by enabling individuals to recognize sabotaging thoughts or behaviors and consciously redirect their attention to more constructive thinking [64]. Our findings suggest that the novel metacognitive awareness of one’s inner critics (saboteurs) may have broadened students’ perceptions of how they were self-sabotaging their relationships with others and themselves, thereby contributing to the observed intervention effects.

Based on existing literature, we also speculate that social connections formed during the “Pod” meetings of the PQ program facilitated greater reflection and introspection. This social interaction likely enhanced participants’ awareness of their inner critics and how these critics negatively impacted them. In a study examining the usefulness of mHealth apps from the perspective of health care workers, Yoon et al [65] found that these workers frequently reported a need for ongoing social support and desired access to an in-app peer support community. According to nudge theory, social influence acts as a significant push factor (nudge) by fostering a sense of belonging to a community with shared goals, thereby enhancing the effectiveness of mHealth apps [65,66]. Therefore, being part of a “Pod” social group likely contributed to sustained engagement in the program, as well as reductions in rumination tendencies and increases in self-compassion. However, because “Pod” group meetings were mandatory and scheduled weekly in this study, future research should explore whether these results are generalizable to settings where “Pod” meetings are nonmandatory and rely on voluntary group participation.

Five months after completing the PQ program, when the “Pod” groups were no longer scheduled, rumination tendencies and self-compassion scores were no longer significantly different from pretest levels. In other words, the PQ program’s effects on rumination tendencies and self-compassion did not persist after 5 months. Given the mechanisms cited for the changes in self-compassion and rumination tendencies immediately after the PQ program, it is not surprising that these effects were not sustained. As the initial insights from becoming aware of one’s self-sabotaging inner critics faded and the “Pod” meetings ceased, the sustained impact on self-compassion and ruminative thinking diminished. However, a more likely explanation for the lack of sustained effects from the PQ program is that a significant percentage of students (46/64, 72%) did not continue using the PQ mobile app regularly after the completion of the 6-week program. Research indicates that it is common for users to engage with mHealth apps only for a short period and that sustained use typically requires clear, ongoing benefits. Approximately 74% of mHealth app users discontinue use by the tenth session [67]. Future studies should explore strategies to motivate health care students to continue benefiting from the PQ app beyond the regular program.

### Practical Implications

Our findings underscore the importance of fostering self-awareness of inner critics to enhance students’ well-being. This core component of the PQ program may have universal applicability across diverse student populations, not just health care students, though further research is needed to confirm this. Encouraging students to engage in practices such as mindfulness and journaling can help them identify and understand their inner critics. Educators can play a crucial role in promoting self-compassion and well-being among students. By being trained in the PQ program, educators can model and cultivate self-awareness in their classrooms. Creating a supportive and nonjudgmental environment allows educators to teach students how to develop self-compassion, challenge negative self-talk, and manage stress effectively. Additionally, integrating principles of self-compassion and well-being into the curriculum can promote lasting awareness and mindfulness, rather than relying solely on a structured program.

### Strengths

This study has several notable strengths. First, it utilized a mindfulness-based PQ training program delivered through a smartphone app, providing a highly scalable, accessible, engaging, and convenient way for participants to regularly access the program, compared with traditional in-person mindfulness classes. Second, the study focused on a specific group of participants: occupational therapy undergraduates. This is a unique population that experiences high levels of stress and could benefit significantly from mindfulness-based interventions. Third, the study used a longitudinal design, enabling us to assess the effects of the intervention over an extended period, up to 5 months after the program concluded. Lastly, the intervention in this study was a 6-week app-delivered PQ program, which has been used with MBA students, athletes, executive teams, and chief executive officers [10]. This novel intervention has not been extensively studied, particularly within the health care student population, making our findings especially valuable.

### Limitation

One limitation of this study is the absence of a comparable intervention group to control for potential confounding factors. Future research should include an active control condition to isolate the specific effects of the PQ program and determine which elements are most effective in enhancing self-compassion and reducing stress and rumination among health care students.

### Conclusions

This study examined the effects of the 6-week PQ program on perceived stress, self-compassion, and rumination tendencies in health care undergraduates. While the findings did not support the hypothesis that the PQ program would reduce perceived stress levels, there were significant increases in self-compassion and reductions in rumination tendencies immediately following the intervention. However, there was no significant effect on self-compassion, rumination tendencies, or perceived stress at the 5-month follow-up, and no evidence that increased daily PQ practice led to greater changes. Our findings suggest that the PQ program helped reframe students’ automatic negative thought processes through mindfulness practice. Additionally, the novel awareness of saboteurs and the weekly “Pod” group meetings may have contributed to the observed intervention effects. Our findings contribute to existing evidence that increased self-acceptance and the practice of withholding judgment toward oneself and others are essential for enhancing self-compassion and reducing rumination. We recommend further research with an active control group to better understand the actual impact of the PQ program and to identify which specific components of the program produce the most significant effects.

## Abbreviations

HSD: honestly significant difference
mHealth: mobile health
PQ: Positive Intelligence
PSS-14: 14-item Perceived Stress Scale
RQ: research question
RRQ-Rum: Reflection Rumination Questionnaire
SCS-SF: Self-Compassion Scale–Short Form

## Data Availability

The data sets generated during or analyzed during this study are available from the corresponding author on reasonable request.
